# DNA methylation changes associated with cannabis use and verbal learning performance in adolescents: an exploratory whole genome methylation study

**DOI:** 10.1038/s41398-022-02025-6

**Published:** 2022-08-06

**Authors:** Melina Wiedmann, Sören Kuitunen-Paul, Lukas Andreas Basedow, Max Wolff, Nataliya DiDonato, Julia Franzen, Wolfgang Wagner, Veit Roessner, Yulia Golub

**Affiliations:** 1 Department of Child and Adolescent Psychiatry, Technische Universität Dresden, Faculty of Medicine, 01307 Dresden, Germany; 2 Technische Universität Chemnitz, Chair for Clinical Psychology and Psychotherapy, Chemnitz, Germany; 3 Charité—Universitätsmedizin Berlin, corporate member of Freie Universität Berlin and Humboldt-Universität zu Berlin, Department of Psychiatry and Psychotherapy, Campus Charité Mitte, Berlin, Germany; 4 Technische Universität Dresden, University Hospital, Institute for Clinical Genetics, Dresden, Germany; 5 Helmholtz-Institute for Biomedical Engineering, Stem Cell Biology and Cellular Engineering, RWTH Aachen University Medical School, Aachen, Germany

**Keywords:** Biomarkers, Human behaviour

## Abstract

The association between extent of chronic cannabis use (CCU-extent) and cognitive impairment among adolescents has been the subject of controversial debate. Linking DNA methylation to CCU-extent could help to understand cannabis associated changes in cognitive performance. We analyzed cognitive task performances, CpG methylation in peripheral whole-blood samples and self-reported past-year CCU-extent of *n* = 18 adolescents (*n* = 9 psychiatric outpatients with chronic cannabis use (CCU), *n* = 9 without) who were matched for age, gender and psychiatric disorders. Patients with CCU were at least 24 h abstinent when cognitive tasks were performed. A Principal Component Analysis (PCA) was carried out to identify group differences in whole genome DNA methylation. Mediation analyses were performed between CCU-extent associated CpG sites and CCU-extent associated variables of cognitive tasks. PCA results indicated large differences in whole genome DNA methylation levels between the groups that did not reach statistical significance. Six CpG sites revealed reduced methylation associated with CCU-extent. Furthermore, CCU-extent was associated with lower scores in verbal learning. All six CpG sites mediated the effects between CCU-extent and verbal learning free recall. Our results indicate that CCU is associated with certain patterns in the methylome. Furthermore, CCU-extent associated impairments in memory function are mediated via differential methylation of the six CCU-associated CpG sits. Six identified CpG are located in genes previously described in the context of neurodegeneration, hippocampus-dependent learning and neurogenesis. However, these results have to be carefully interpreted due to a small sample size. Replication studies are warranted.

## Introduction

Global perception of cannabis use has been going through remarkable changes during the past years, with the public perceiving cannabis as less and less harmful [1]. Especially adolescents tend to report low perceived harm of cannabis and show increasing rates of consumption [2]. Prevalence rates of 19% for last year cannabis use are being reported among adolescents and young adults (15–24 years) [3]. Especially in the period of adolescence which involves drastic neurobiological and behavioral changes, e.g., myelination and maturation of prefrontal regions [4–7] the effect of chronic cannabis use (CCU) on the modulation of cognitive performance has to be thoroughly investigated. Thus, several studies demonstrated cannabis exposure being associated with lower academic performance [8, 9], intelligence quotient decline [10, 11], and lower cognitive performance in adolescents [8, 10].

Verbal learning and memory impairments in adolescent CCU is a robust finding [12, 13]. Numerous laboratories have demonstrated verbal learning and memory decline in adult and adolescent CCU [14–19]. However, neuromolecular mechanisms of CCU associated learning and memory decline are not known.

Cannabinoid signaling in the brain is mainly mediated by the cannabinoid receptor 1 (CB1R) [20] the most abundant G protein-coupled receptor in the mammalian brain widely expressed in the basal ganglia, cerebellum, hippocampus, and prefrontal cortex [21, 22]. Experimental studies showed that exposure to THC (Δ^9^-trans-Tetrahydrocannabinol; the main psychoactive compound in cannabis) goes along with an increase in *CNR1* (CB1R) mRNA levels in rats, which was accompanied by symptoms of cognitive impairments [23]. Cannabis-induced over-activation of CB1R expression has been reported to suppress long-term potentiation in apical dendrites of CA1 pyramidal neurons of the hippocampus [24]. Long-term potentiation might play an important role in maintaining memory contents [25] thus proposing a mechanism by which THC exposure could affect learning and memory functions. In support of this theory, high frequency CCU has been associated with lower left hippocampal volume, who has been shown to mediate the relationship between CCU and impairments in working memory in adults [26].

Changes in gene expression are largely regulated by the epigenome, particularly DNA methylation, which is responsive to environmental input and can directly induce persistent patterns of gene activation/inactivation impacting the phenotype [27]. Exposure to THC has been shown to affect whole genome DNA methylation in cannabis using women [28]. Further, cannabis and THC exposure might affect CB1R expression [29] as well as neurodevelopment and neuronal signaling [30–32] via CpG methylation changes.

Our study aims for a better understanding of the biological pathways underlying cognitive consequences of CCU. Specifically, we are interested in the role of epigenome i.e., CCU induced DNA methylation changes in cannabis associated verbal learning and memory impairments in adolescents. In the first step, we compared the whole genome DNA methylation levels in adolescents with and without CCU to replicate previous results that indicated an association between lifetime cannabis use and decreased whole genome DNA methylation [28]. In the second step we identified cognitive variables and differentially methylated CpG sites associated with extent of chronic cannabis use (CCU-extent). In order to test the hypothesis that the relationship between CCU-extent and cognitive task performance is exerted by CpG methylation, mediation analyses were performed. Given that both alcohol and nicotine are frequently used in our study sample [33] and may affect CpG methylation as well [34, 35], the use of these substances had to be controlled for in our analyses. Furthermore, we used time since last cannabis use as another control variable, since both memory function and dynamic changes in DNA methylation can be affected by short term cannabis exposure.

## Material and methods

### Study design

Data for the current analyses were collected from adolescents who applied to the outpatient department for adolescent substance abuse, C. G. Carus University hospital, Dresden, Germany. Participants and their legal guardians were informed about the study at the first consultation appointment and gave their written informed consent. All procedures were approved by the Institutional Review Board of the C. G. Carus University hospital, Dresden (EK 66022018) and registered at clinicaltrials.gov (NCT03444974; details see in Supplementary Information).

### Participants

After excluding participants who did not meet inclusion criteria, *n* = 9 individuals with CCU were included for DNA methylation analyses.

**Fig. 1:**
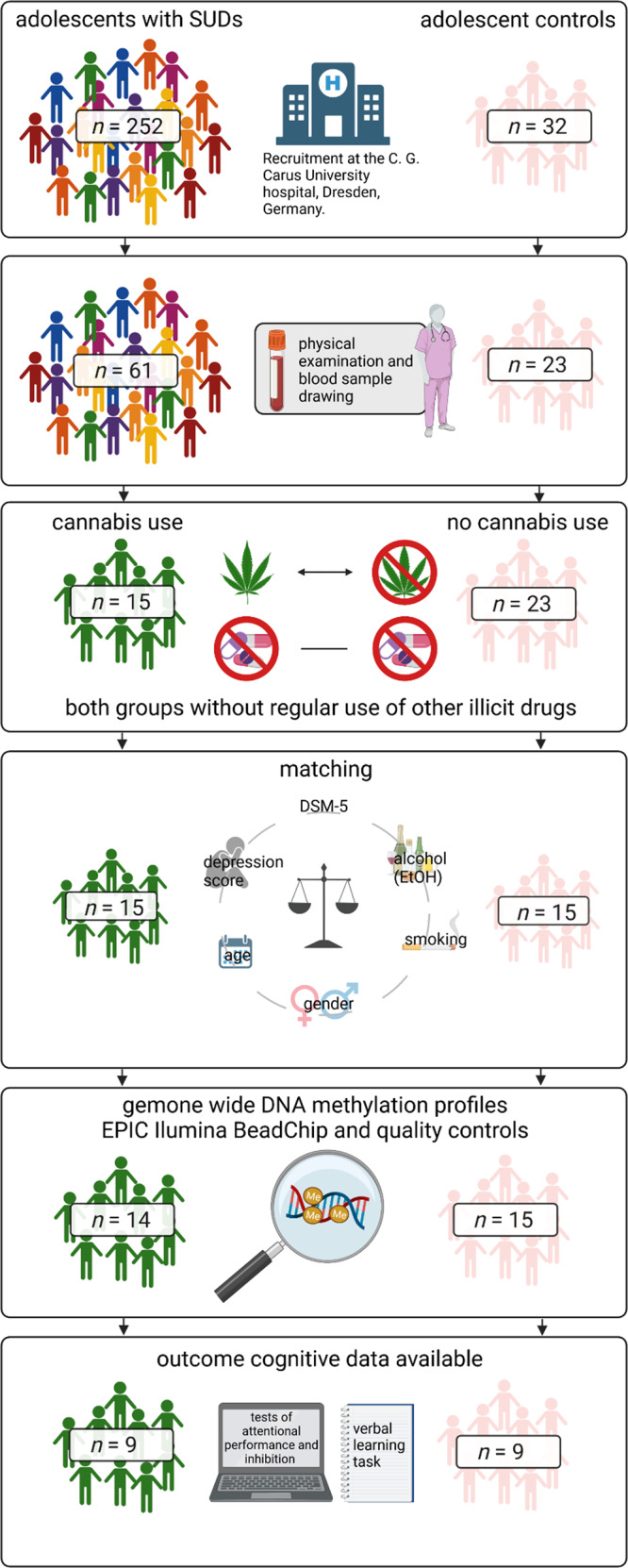
Process of sample composition and course of study. The figure was created with BioRender.com.

Additionally, we included *n* = 9 individuals with no lifetime use of illicit substances as controls, after matching them for age, gender, alcohol and tobacco use and co-occurring psychiatric disorders, see Fig. 1 and Table 1.

**Table 1:** Characteristics of demographics, substance use and comorbid disorders of the sample.

	CCU group (*n* = 9)	Controls without CCU (*n* = 9)	CCU group vs. Controls without CCU		
	*M* (SD)/*N* (%)		*F*(1,15)	η^2^_part_	*p*
Cannabis use frequency^a^	21.0 (10.9) days	0.0 (0.0) days			
Possible covariates for mediation analysis					
Tobacco use frequency^a^	24.2 (9.2) days	0.1 (0.3) days	58.05	0.79	<0.001
Alcohol use frequency^a^	5.9 (9.9) days	0.1 (0.3) days	2.84	0.16	0.113
Male gender	7 (77.8%)	7 (77.8%)	0.02	<0.01	0.901
Age	16.0 (1.3) years	15.2 (1.7) years	0.76	0.05	0.397
BDI-II score	9.3 (7.6)	10.9 (12.3)	0.09	0.01	0.764
Cannabis Dependence (ICD-10)	6 (66.7%)	0 (0%)			
Cannabis harmful use (ICD-10)	3 (33.3%)	0 (0%)			
Number of co-occurring psychiatric disorders^b^	0.9 (1.1)	0.9 (1.6)	<0.01	<0.01	0.984

*N* = 1 reported MDMA use with 4 days of use per month. *N* = 1 reported abuse of prescribed drugs and was 9 months abstinent at the time of taking blood samples). For descriptive table using quantity-frequency indices for substance use variables, see Table 2.

*BDI-II* Beck’s Depression Inventory version 2.

^a^average days per month during previous year.

^b^excluding Substance Use Disorders.

We successfully matched the two groups on gender, age, BDI-II scores (depressive symptoms) and number of co-occurring disorders. Since we were not able to sufficiently match the groups on tobacco and alcohol use frequency, we performed mediation analyses including tobacco and alcohol use frequency as covariates to test for confounding influences (details see in Supplementary Tables S1–4).

### DNA methylation analysis

DNA was isolated from whole blood samples using the Qiagen Blood Mini kit (Qiagen, Hilden, Germany) and sent to Life & Brain GmbH (Bonn, Germany) for bisulfite conversion and subsequent analysis on the Illumina Infinium Methylation EPIC Bead Chip. Beta values were calculated after quantile normalization which range from 0 to 1 and reflect DNA methylation levels for each CpG site (details see in Supplementary Information).

### Cognitive testing

Participants performed six cognitive tests with 10 different test outcomes. The six tests can be categorized as following: tests of verbal learning, tests of attentional performance (TAP divided attention and TAP alertness) and tests of inhibitory control (Test of Attentional Performance (TAP) Go/NoGo, Stroop, and Stop-Signal).

### Verbal learning

We used the manualized Verbal Learning and Memory Test (VLMT) [36] in which participants were presented a word list out five times (trial 1–5), which participants had to recall (free recall trial). After an interference list and a 20-min break, participants had to recall the first list again. Finally, participants had to indicate words from the first list among a list containing target words and distractors. We chose 3 variables which included learning ability (*free recall trial*), long-term consolidation (*loss after temporal delay*) and long-term recognition performance (*cued recall trial;* details see in Supplementary Information).

### Attentional performance

We used the Test of Attentional Performance (TAP) [37] to measure attentional performance: “alertness” which consists of two subsets (cued and non-cued) and “divided attention” (details see in Supplementary Information).

### Inhibitory control

We used the “Go/NoGo” task of the TAP and a number Stroop task and a Stop-Signal task [38, 39] implemented using the Psychophysics Toolbox in MATLAB to measure inhibitory control (details see in Supplementary Information).

The final analysis of cognitive testing included the following variables: (1) Stroop Task, (2) Stop-Signal Task, (3) TAP Go/NoGo, (4) Divided Attention - auditory, (5) Divided Attention - visual, (6) Alertness - cued, (7) Alertness - non-cued, (8) VLMT - free recall trial, (9) VLMT loss after temporal delay and (10) VLMT cued recall trial.

### Substance use

The extent of substance use was measured as the average amount of use per usual consumption day multiplied with average frequency of use during previous year:

$$Extent\,of\,Substance\,use = Q \times F$$

Q = Average quantity per day of use during previous year in gram.

F = Frequency during previous year in days per month.

CCU was defined as at least weekly use of cannabis during the previous year and cannabis use related problems (details see in Supplementary Information).

### Qualitative measures of THC Consume

Abstinence was monitored via urine screening before testing cognitive task performance, all individuals were required to be at least 24 h abstinent when cognitive testing was performed (details see in Supplementary Information).

### Statistical analysis

A Principal Component Analysis (PCA) was performed to test for group differences in whole genome DNA methylation. A subsequent analysis of variances (ANOVA) was performed with whole genome DNA methylation as dependent and group as independent variable.

We performed a Pearson correlation analysis between CCU-extent CpG sites and the results of cognitive testing, thus identifying thus identifying CCU-extent associated CpG sites and cognitive variables. Our results were corrected for alpha inflation. Normal distribution was met for the scores of the VLMT free recall trial, beta values of the *k* = 6 identified CpGs, CCU-extent and the scores of the VLMT cued recall trial were not normally distributed.

Further, we carried out a Multivariate Analysis of Variance (MANOVA) with the results of the cognitive tasks as a dependent variable and group (CCU group vs. controls without CCU) as an independent variable to test for differences between the groups. We performed a mediation analysis between CpG sites previously associated with CCU extend and CCU-extent associated cognitive variables.

Further, we performed two posteriori analyses to exclude possible confounding variables. First, we performed mediation analyses with frequency of tobacco and alcohol use as covariates with revealed no influence on the mediation models, for further details please see Supplementary Tables S1–4.

Second, we performed a MANOVA with scores in VLMT free recall trial and VLMT cued recall trial as dependent variable and time since last THC exposure as independent variable in order to control for short term and possible withdrawal effects. The results revealed no differences in scores of VLMT free recall nor in VLMT cued recall trial between current and abstinent users (please see details in the Supplementary Table S9).

## Results

### Lower whole genome DNA methylation levels in adolescents with CCU

**Fig. 2:**
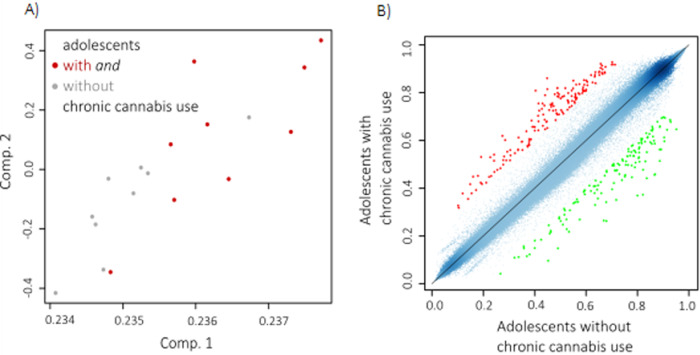
DNA methylation changes upon adolescents with chronic cannabis use. **A** Scatter plots of PCA results of *n* = 9 adolescents with CCU and *n* = 9 without CCU displaying factor load of whole genome DNA methylation (beta values) of *k* = 846,604 CpG sites after removing X/Y chromosomes on two principal components (Comp.1 and Comp.2). **B** Scatter plot of mean methylation levels (beta values) for each CpG of adolescents with CCU (*n* = 9) and without CCU (*n* = 9). CpG sites differing in their methylation levels with more than 20% are highlighted in green (hypomethylation, *k* = 50) and red (hypermethylation, *k* = 61).

The PCA indicated that whole genome DNA methylation profiles might differ in adolescents with *versus* without chronic cannabis use (Fig. 2A). The ANOVA with whole genome DNA methylation as outcome and group as predictor revealed a large, however non-significant difference in whole genome DNA methylation between adolescents with CCU (*M* = 61.7^−2^, *SD* = 7.9^−4^) and controls (*M* = 61.8 10^−2^, *SD* = 6.8^−4^), (*F*(1,17) = 3.33, *p* = 0.087, η^2^_part_ = 0.17). *K* = 61 CpG sites showed a hypermethylation in adolescents with CCU and *k* = 50 CpG sites showed a hypomethylation with differences of >20% in methylation status between adolescents with CCU and controls (Fig. 2B).

### No group differences in cognitive tasks

In order to test whether the groups differ in their cognitive performance in general which could affect the relationship between CCU-extent and cognitive variables, we performed an ANOVA with cognitive variables as dependent variable and group as independent variable. We found no differences between the groups (see Table 3).

### Adolescents with higher extent of chronic cannabis use reveal lower scores in verbal learning tasks

In order to identify cognitive variables associated with CCU-extent we performed correlation analyses for *n* = 10 cognitive variables. Two Verbal Learning and Memory Test (VLMT) variables met criteria for further testing (at least medium sized correlations, neglecting level of significance due to small sample size). These variables showed medium to large correlations with CCU-extent (see Table 4) i.e., a medium-sized non-significant correlation for the VLMT free recall trial (*r* = −0.36, *p* = 0.145) and a large association for cued recall (*r* = −0.60, *p* = 0.008). This indicates that CCU-extent was associated with reduced verbal learning ability indicated by lower scores in both the VLMT free recall trial and VLMT cued recall.

### Higher CCU-extent is associated with hypomethylation in six CpG sites

In the next step, we performed correlation analyses between CCU-extent and *k* = 866238 CpG sites to identify CCU-extent associated CpG sites for further mediation analyses. After adjusting for multiple testing, methylation of *n* = 6 CpG sites (Cg17285328 related to gene SH3 And PX Domains 2B *(SH3PXD2B)*; Cg20777378, intergenic CpG; Cg04904300, related to gene DGCR8 Microprocessor Complex Subunit (*DGCR8*); Cg08923376, related to gene Zinc Finger Protein 107 *(ZNF107)*; Cg04270414, related to genes Sorting Nexin 15 (*SNX15*) and SAC3 Domain Containing 1 *(SAC3D1);* Cg23767840, related to gene Epsin 2 (*EPN2*) was associated with CCU-extent (*r* = −0.93 to −0.95; *p* = 3.78^−8^ to 1.20^−9^). All CCU-extent associated CpG sites were negatively correlated with CCU-extent. Patients with very high cannabis use revealed the lowest beta values in these *k* = 6 CpGs, supporting our results on the cannabis effect with regards to gene methylation.

In order to test the associations of CCU-extent and hypomethylation of the identified CpG sites in a larger sample, we repeated the correlation analysis in an extended sample of *n* = 29. Similar to our findings with *n* = 18, higher CCU-extent was associated with hypomethylation in the *k* = 6 CpG sites, with reduced but still large effect sizes (*r* = −0.52 to −0.71, *p* = 0.004 to 1.51E-05), please see Supplementary Table S10.

### CCU-extent associated impairments in VLMT free recall trial are mediated via differential methylation of six a CpG sits located in SH3PXD2B, DGCR8, ZNF107, SNX15/SAC3D1 and EPN2 genes

In order to test whether the relationship between CCU-extent and cognitive task performance is exerted by CpG site methylation we carried out *k* = 12 mediation analyses, for *k* = 2 CCU-extent associated cognitive variables and for *k* = 6 CCU-extent associated CpG sites. The mediation analyses for VLMT free-recall showed indirect effects for all six CpG sites (*Z* = −2.01 to −2.96; *p* = 0.045 to 0.004) while explaining most of the total variance (*R²* = 85.6% to 90.6%), see Table 5. Therefore, the relationship between lower performance in VLMT free recall trial and high CCU-extent was due to CCU-extent associated hypomethylation of CpGs further affecting scores in VLMT free recall trial.

The results of the mediation analyses for the association between CCU-extent and scores in VLMT cued recall trial revealed no mediating effects (*Z* = −0.63 to −0.94; *p* = 0.346 to 0.528, see Table 6), with total variances explained by each model ranging from 85.6% to 90.6%.

### Gender influences

To control for gender bias, we removed CpGs of X/Y chromosomes from the analysis and matched age and gender in our sample groups. Further, we performed a binary regression analysis between gender and CCU-extent, scores of the VLMT free recall and cued recall trials, and the methylation of the CpG sites located in the *SH3PXD2B, DGCR8, ZNF107, SNX15/SAC3D1*, and EPN2 genes. Results of the binary regression analysis revealed that gender did not influences these variables, please see Supplementary Table S11.

## Discussion

This explorative study investigated whether adolescents with and without CCU differ in cognitive performance and genome wide DNA methylation levels. Furthermore, we aimed to investigate whether CCU-extent is associated with methylation changes at single CpG sites and whether differential CpG methylation mediates the relationship between CCU-extent and cognitive task performance in a sample of adolescents with and without CCU.

Despite the restricted number of subjects and conservative corrections for multiple testing, we could show decreased whole genome DNA methylation profiles in adolescents with CCU compared to adolescents without CCU.

We identified six CpG sites (*SH3PXD2B*, *DGCR8*, *ZNF107, SNX15/SAC3D1,* and *EPN2*) that mediated the relationship between CCU-extent and verbal learning performance in a free recall trial of a verbal learning task.

Our results can be cautiously interpreted as the first evidence that the relationship between cannabis use and verbal learning could be translated via changes in methylation of specific CpG sites, located at *SH3PXD2B*, *DGCR8*, *ZNF107, SNX15/SAC3D1,* and *EPN2* genes. One of these CpG sites (cg20777378) is not positioned in a proximity to a certain gene. Notably, all six genes (*SH3PXD2B, DGCR8*, *ZNF107*, *SNX15/SAC3D1,* and *EPN2*) have previously been associated with neurodegeneration, hippocampus-dependent learning and neurogenesis, as outlined below. To our knowledge, this is the first study linking cannabis, verbal learning performances, and DNA methylation.

### Whole genome DNA methylation

Our results show decreased whole genome DNA methylation levels in adolescents with CCU compared to controls. Our findings are in line with a previous study indicating that THC exposure leads to a loss of whole genome methylation in granulosa cells of cannabis using women [28]. This relationship proposedly acts via reduced DNA methyltransferase (DNMT) expression possibly inhibiting the cells ability to maintain epigenetic integrity and thus reducing DNA methylation [28]. We do not know how stable these methylation changes are, however, it is possible that they persist and be transmitted over generations [40, 41].

### Cognitive task performance

Contrary to our expectations, we did not see differences in cognitive task performance between the groups. This might be due to the fact that we partially included patients that were abstinent from cannabis during the previous weeks which might have ameliorated the effects of cannabis use on cognitive task performance at the time of testing and were not detectable in our small sample. A previous study indicated that 4 weeks of abstinence might already ameliorate cannabis associated impairments in cognitive task performance [42]. The two groups only differed in cannabis, tobacco and alcohol use and the fact that we only saw an association with CCU-extent and no group differences seems to underline the impact on CCU-extent and not presence of regular cannabis in general on cognitive performance.

The correlation between CCU-extent and impaired verbal learning in our sample is in line with previous findings indicating an association between slower verbal learning and adolescent cannabis use [43]. One recent meta-analysis found small differences in delayed memory and learning between cannabis using adolescents and non-using controls [44]. In our sample, we observed an association between CCU-extent and impaired cognitive performance in overall verbal learning performance (VLMT free recall) and cued recall after temporal delay which are in line with the findings from a previous study [44].

### Extent of chronic cannabis use and CpG methylation

**Fig. 3:**
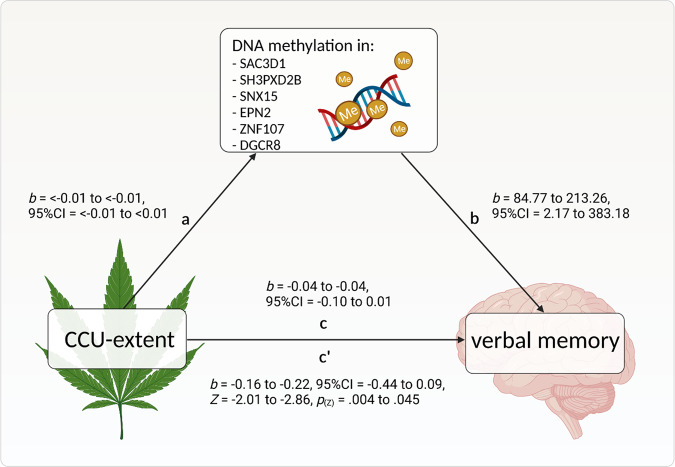
Mediation effects of six CCU-associated CpG sites on the relationship between CCU-extent and verbal memory. CCU-extent associated impairments in verbal learning (scores in VLMT free recall trial) are mediated via differential methylation patterns of the six CCU-associated CpG sites in *SH3PXD2B* (Cg17285328), *DGCR8* (Cg04904300), *ZNF107* (Cg08923376), *SNX15;SAC3D1 (*Cg04270414), *EPN2* and (Cg23767840) genes. Effect sizes for path a: *b* = <0.01 to <0.01, path b: *b* = 84.77 to 213.26, path c: *b* = −0.04 to −0.04 and path c’: *b* = −0.16 to −0.22, *p*_(Z)_ = 0.004 to 0.045. Please see Tables 5 and 6 for further details. The figure was created with BioRender.com.

Hypomethylation on six CpG sites was associated with CCU-extent, which was still prevalent after testing for possible confounders: tobacco and alcohol use, as well as and duration of abstinence, see Fig. 3. However, there are other variables that define the impact of CpG methylation on the gene expression levels, e.g., location in reference to promotor or enhancer region [45, 46]. Unfortunately, we were not able to measure RNA and protein levels in our samples. Therefore, we can only speculate about consequences of the methylation changes in the identified CpG sites on DNA transcription and protein synthesis.

Interestingly, three of these genes (*EPN2*, *SNX15,* and *DGCR8*) have previously been associated with hippocampal functioning and processes linked to the development of Alzheimer’s disease (AD). AD is a mental disorder that is mainly characterized by loss of memory [47]. Increased *EPN2* and *SNX15* expression have previously been linked to AD’s neurodegenerative processes. High levels of epsin 1 and 2 (encoded by *EPN2*) have been shown to be fundamental in the signaling of the notch pathway [48]. Notch signaling may contribute to neurodegeneration and has been associated with the advancement of Alzheimer’s disease [49], increased Amyloid β aggregation and apoptosis [50, 51].

*DGCR8* has also been associated with brain regions involved in memory formation e.g., the hippocampus. However, our results are contrary to previous findings on *DGCR8* expression and learning ability. Our results reveal a negative relationship between *DGCR8* expression (indicated by low methylation levels of *DGCR8*) and verbal learning performance and are contrary to previous findings indicating a positive relationship between *DGCR8* expression and learning ability. Reduced *DGCR8* expression has been shown to impair cell proliferation and neurogenesis in hippocampus as well as hippocampus-dependent learning in mice [52]. Future studies are needed to shed light on this topic and to examine the relationship between *DGCR8* expression and learning ability.

*SH3PXD2B* and *ZNF107* expression have also been previously connected with neurogenesis via regulation of small organelles, so called cilia, which are involved in mechanisms in neural development [53] and regulation of neuronal cell death [54]. Zink finger protein 107 (*ZNF107*) and SH3 and PX domain-containing protein 2B (*SH3PXD2B)* have been shown to be required for ciliogenesis [53, 54] possibly affecting learning ability.

Our results imply that CCU-extent in adolescents with CCU is associated with changes in DNA methylation in genes that have previously been associated with neurodegeneration, hippocampus-dependent learning and neurogenesis. Importantly, transgenerational transmission is common for methylation changes [40, 41], thus THC exposure may alter methylation patterns not only in the exposed individuals but also in the subsequent generations leading to an increased risk for memory impairments and/or other cognitive alterations.

### Limitations

Our exploratory study has several limitations: First, the sample size of our study is small which constrains the generalizability of our results. Nonetheless, small sample sizes are common in studies with outpatient adolescents with CCU and to our knowledge, there is no previous study on genome wide DNA-methylation changes associated with cannabis consume in adults and/or adolescents. Furthermore, we carefully controlled for confounding variables e.g., comorbid substance use that is often neglected in studies, and applied careful matching procedures. Considering the fact that this is the first study linking adolescent cannabis use with methylation changes and cognitive performance we believe that our results are a valuable contribution as it may stipulate future studies.

Second, it is important to note, that we can only assume that changes in the methylation of the identified CpG sites might occur in adolescents with CUD and not in adolescents that use cannabis without meeting the criteria for CUD. Therefore, the observed associations between CCU-extent and DNA methylation refer to extreme users with significant impact of cannabis use on health and general functioning. Importantly, the lowest DNA-methylation was observed in patients with very high cannabis consume thus depicting the effect of high cannabis exposure.

Third, due to the cross-sectional design of this study, we cannot draw causal conclusions. Future studies should apply longitudinal design to examine inter-individual changes in methylation patterns. One promising approach could be analyzing individuals before and after achieving abstinence from cannabis.

Fourth, since THC can still be detected weeks after abstinence especially in long term users [55], we were not able to record the exact time of abstinence since cannabis was previously consumed. However, we only included individuals that were abstinent at least 24 h at the time when cognitive tasks were performed, the observed results cannot be due to acute intoxication. Additionally, we controlled for the impact of positive THC drug screening (rest THC values, for further details please see Supplementary Information) and failed to detect differences in verbal learning task performance. Therefore, we assume that the effect of abstinence does not affect verbal learning task performance in our sample.

Fifth, despite the additional mediation analyses with alcohol and nicotine exposure as control variables our small sample size may not be able to detect small effects of nicotine and alcohol. DNA methylation patterns associated with cognitive deficits have previously been associated with adolescent alcohol [56] and nicotine [57] exposure, however, none of the six identified genes in our sample has previously been associated with nicotine or alcohol exposure in terms of DNA methylation.

## Conclusion

This is the first explorative analysis assessing CpG methylation in adolescent cannabis users with cannabis use disorder and linking CCU-extent associated methylation to verbal learning impairment. We showed that cannabis use was associated with CpG methylation changes of genes that have previously been associated with neurodegenerative processes and hippocampus-dependent learning.

### Supplementary information

Supplementary Material

## Appendix

Tables 2–6

**Table 2:** Characteristics of demographics, substance use and comorbid disorders of the sample.

	CCU group (*n* = 9)	Controls without CCU (*n* = 9)	CCU group vs. Controls without CCU		
	*M* (SD)/*N* (%)		*F*(1,15)	*p*	η^2^_part_
Cannabis use QF^b^	21.0 (10.9)	0.0 (0.0)			
Possible covariates for mediation analysis					
Male^a^	7 (77.8%)	7 (77.8%)	0.02	0.901	<0.01
Age^b^	16.0 (1.3)	15.2 (1.7)	0.76	0.397	0.05
Tobacco use QF^b^	24.2 (9.2)	0.1 (0.3)	58.05	<0.001	0.79
Alcohol use QF^b^	5.9 (9.9)	0.1 (0.3)	2.84	0.113	0.16
BDI-II Score^b^	9.3 (7.6)	10.9 (12.3)	0.09	0.764	0.01
Cannabis dependence (ICD-10)	6 (66.7%)	0 (0%)			
Cannabis harmful use (ICD-10)	3 (33.3%)	0 (0%)			
Number of comorbid psychiatric disorders^c^	0.9 (1.1)	0.9 (1.6)	<0.01	0.984	<0.01

QF = Quantity - Frequency Index *n* = 1 reported MDMA use with 4 days of use per month. *n* = 1 Person reported abuse of prescribed drugs and was 9 month abstinent at the time of taking blood samples).

*BDI-II* Beck’s depression Inventory.

^a^categorical variable.

^b^continuous variable.

^c^excluding Substance Use Disorders.

**Table 3:** Characteristics of cognitive performance of the sample.

	CCU group (*n* = 9)	Controls without CCU (*n* = 9)	CCU group vs. Controls without CCU		
	*M* (SD)		*F*(1,15)	*p*	η^2^_part_
Inhibitory control (reaction time in ms)					
Stroop	145.2 (118.5)	166.2 (133.1)	0.13	0.728	0.01
Stop-Signal	165.8 (41.2)	186.6 (55.2)	0.82	0.378	0.05
Go/NoGo	388.7 (62.8)	383.8 (55.8)	0.03	0.864	<0.01
Divided Attention (reaction time in ms)					
Auditory	601.9 (64.8)	584.3 (101.4)	0.19	0.667	0.01
Visual	784.6 (118.9)	815.2 (122.9)	0.29	0.599	0.02
Alertness (reaction time in ms)					
Cued	239.8 (43.1)	248.2 (21.6)	0.27	0.061	0.02
Non-cued	247.3 (46.1)	269.6 (31.6)	1.44	0.248	0.08
VLMT (number of correct words)					
Free recall trial (no delay)	50.7 (12.4)	48.3 (7.3)	0.24	0.634	0.01
Loss after temporal delay (temporal delay)	13.1 (2.1)	13.7 (1.1)	0.50	0.492	0.03
Cued recall trial (temporal delay)	1.6 (1.4)	1.3 (1.6)	0.10	0.758	0.01

**Table 4:** Pearson Correlations *r* (*p*-value) between self-reported cannabis use and cognitive tasks.

		Test of Attentional Performance (TAP)						Verbal Learning Memory Task (VLMT)		
	Inhibitory control			Divided Attention		Alertness		No delay	Temporal delay	
	Stroop	Stop-signal	Go/NoGo	Auditory	Visual	Cued	Non-cued	Free recall trial	Loss after temporal delay	Cued recall trial
Extent of chronic cannabis use	−0.08	−0.14	0.09	−0.04	0.07	−0.18	−0.21	−0.36	0.12	−0.60
	(0.755)	(0.570)	(0.718)	(0.862)	(0.768)	(0.469)	(0.399)	(0.145)	(0.631)	(0.008)

**Table 5:** Mediating effects of CpG methylation for association between self-reported cannabis use and VLMT free recall trial.

CpG site	*Gene*	Position Assembly CRCh37/hg19		*a*		*b*		*c*	*c‘*	Sobel test	
			*R²*	b^a^ [95% CI]	*R²*	b^a^ [95% CI]	*R²*	b^a^ [95% CI]	b^a^ [95% CI]	Z	*p*_(Z)_
				<−0.01		213.26		−0.04	−0.22		
Cg17285328	*SH3PXD2B*	Chr5:171834167	90.6%	[<−0.01, <−0.01]	44.0%	[43.33, 383.18]	12.8%	[−0.10, 0.01]	[−0.41, 0.01]	−2.49	0.013
				<−0.01		128.11		−0.04	−0.16		
Cg20777378	*–*	Chr3:184387942	88.2%	[<−0.01, <−0.01]	36.2%	[17.55, 238.67]	12.8%	[−0.10, 0.01]	[−0.31, 0.06]	−2.20	0.028
				<−0.01				−0.04	−0.18		
Cg04904300	*DGCR8*	Chr22:20076641	87.5%	[<−0.01, <−0.01]	42.7%	127.28 [42.62,211.94]	12.8%	[−0.10, 0.01]	[−0.34, 0.02]	−2.86	0.004
				−0.01		84.77		−0.04	−0.16		
Cg08923376	*ZNF107*	Chr7:64149928	87.4%	[<−0.01, <−0.01]	37.5%	[2.17, 167.38]	12.8%	[−0.10, 0.01]	[−0.32, <−0.01]	−2.01	0.045
				−0.01		117.57		−0.04	−0.16		
Cg04270414	*SNX15; SAC3D1*	Chr11:64807235	86.9%	[<−0.01, <−0.01]	38.1%	[10.90, 224.23]	12.8%	[−0.10, 0.01]	[−0.30, 0.09]	−2.10	0.036
				−0.01		104.47		−0.04	−0.17		
Cg23767840	*EPN2*	Chr17:19174122	85.6%	[<−0.01, <−0.01]	41.8%	[11.70, 197.25]	12.8%	[−0.10, 0.01]	[−0.44, 0.02]	−2.13	0.033

^a^unstandardized coefficient b. *a* displays the relationship between predictor and mediator. *b* displays the relationship between mediator and outcome variable. *c* displays the relationship between predictor and outcome variable. *c’* displays the indirect predictor and mediator.

**Table 6:** Mediating effects of CpG methylation for association between self-reported cannabis use and VLMT cued recall trial.

CpG site	*Gene*	Position Assembly CRCh37/hg19		*a*		*b*		*c*	*c‘*	Sobel Test	
			*R²*	b^a^ [95% CI]	*R²*	b^a^ [95% CI]	*R²*	b^a^ [95% CI]	b^a^ [95% CI]	Z	*p*_(Z)_
				<−0.01		9.83		−0.01	−0.01		
Cg17285328	SH3PXD2B	Chr5:171834167	90.6%	[<−0.01, <−0.01]	38.7%	[−14.73, 34.40]	36.2%	[−0.02, −0.01]	[−0.03, 0.02]	−0.84	0.401
				<−0.01		9.49		−0.01	−0.01		
Cg20777378	–	Chr3:184387942	88.2%	[<−0.01, <−0.01]	41.0%	[−11.22, 30.20]	36.2%	[−0.02, −0.01]	[−0.03, 0.02]	−0.94	0.346
				<−0.01		6.06		−0.01	−0.01		
Cg04904300	DGCR8	Chr22:20076641	87.5%	[<−0.01, <−0.01]	38.8%	[−14.11, 26.23]	36.2%	[−0.02, <−0.01]	[−0.03, 0.02]	−0.63	0.528
				<−0.01		5.76		−0.01	−0.01		
Cg08923376	ZNF107	Chr7:64149928	87.5%	[<−0.01, <−0.01]	40.4%	[−12.59, 24.11]	36.2%	[−0.02, −0.01]	[−0.03, 0.01]	−0.65	0.513
				<−0.01		7.68		−0.01	−0.01		
Cg04270414	SNX15; SAC3D1	Chr11:64807235	86.9%	[<−0.01, <−0.01]	40.2%	[−10.20, 25.56]	36.2%	[−0.02, <−0.01]	[−0.03, 0.02]	−0.88	0.376
				<−0.01		5.77		−0.01	−0.01		
Cg23767840	EPN2	Chr17:19174122	85.6%	[<−0.01, <−0.01]	39.5%	[−10.43, 21.98]	36.2%	[−0.02, −0.01]	[−0.03, 0.02]	−0.74	0.461

^*a*^unstandardized coefficient b. *a* displays the relationship between predictor and mediator. *b* displays the relationship between mediator and outcome variable. *c* displays the relationship between predictor and outcome variable. *c’* displays the indirect predictor and mediator.
