# Supplementary material for: DNA methylation changes associated with cannabis use and verbal learning performance in adolescents: an exploratory whole genome methylation study
Source: Transl Psychiatry. 2022 Aug 6;12:317. doi: 10.1038/s41398-022-02025-6 (PMC9357061; doi:10.1038/s41398-022-02025-6)
Supplement: Supplementary file 1 — Supplementary Material [file 41398_2022_2025_MOESM1_ESM.docx]

Supplemental Information

*Study Design*

This study is part of a larger study registered at clinicaltrials.gov (NCT03444974), at the C. G. Carus University hospital, Dresden, that evaluates an outpatient group-based intervention programme for adolescents with chronic substance use ^1^. Data for the current analyses were collected from the participants from November 2017 until July 2020. Data collection was embedded into the standard procedures of the diagnostic processes of the outpatient department, including appointments for cognitive testing and physical examination. Participants and their legal guardians who applied to the outpatient department for adolescent substance abuse, C. G. Carus University hospital, Dresden, Germany, were informed about the study at the first consultation appointment and gave their written informed consent. All procedures were approved by the Institutional Review Board of the C. G. Carus University hospital, Dresden (EK 66022018) and registered at clinicaltrials.gov (NCT03444974).

*Participants*

Out of *n* = 252 participants, *n* = 61 participants agreed to give blood samples. Of these *n* = 46 participants were excluded due to either use of illicit substances other than cannabis during previous month, regular illicit substance use other than cannabis during previous year (more than weekly use) or any form of chronic medication. *N* = 1 was removed due to insufficient data quality, leaving *n* = 14 individuals for DNA methylation analyses. For mediation analyses with cognitive task performance further *n* = 5 had to excluded due to missing data on cognitive task performance.

Additionally, we included *n* = 25 adolescents with no lifetime use of illicit substances as controls. They included patients recruited from general child & adolescent psychiatric departments at our clinic as well as non-patients that were recruited via local advertisements for study participation. *N* = 23 out of *n* = 25 controls agreed to give blood samples. The control group was matched for age, gender, alcohol and tobacco use and co-occurring psychiatric disorder classes (psychotic disorders, mood disorders, anxiety disorders, conduct disorders, obsessive compulsive disorders, posttraumatic stress disorders, eating disorders and tic disorders) which led to exclusion of *n* = 14 leaving *n* = 9 controls for mediation analysis, see Figure 1 and Table 1.

The mean age across groups was *M* = 15.6 (*SD* = 1.6) years with mainly male participants included (*n* = 14, 77.8%), see Table 1. We successfully matched the two groups on gender, age, BDI-II score (depressive symptoms) and number of co-occurring disorders. Since we were not able to sufficiently match the groups on tobacco and alcohol use frequency, we performed mediation analyses including tobacco and alcohol use frequency as covariates to test for confounding influences.

*DNA methylation analysis*

Blood samples were drawn from participants in the morning, with participants being asked to abstain from breakfast. The samples were archived at –80°C. DNA was isolated from 1.8 – 2.0 ml whole blood samples using the Qiagen Blood Mini kit (Qiagen, Hilden, Germany) and 1.2 µg were sent to Life & Brain GmbH (Bonn, Germany) for bisulfite conversion and subsequent analysis on the Illumina Infinium Methylation EPIC Bead Chip. DNA methylation analysis was performed by Cygenia GmbH (Aachen, Germany). Beta values at approximately 850,000 CpGs were calculated after quantile normalization using the R package minfi ^2^ which range from 0 to 1 and reflect DNA methylation levels for each CpG site. Quality controls were performed using probe intensities, beta value distributions, unsupervised clustering as well as age and gender predictions, leading to the exclusion of one sample. CpG sites of the X and Y chromosomes were excluded from further analysis to prevent gender bias.

*Verbal learning*

We used the manualized Verbal Learning and Memory Test (VLMT, Helmstaedter et al., 2001) which is a German version of the Rey Auditory Verbal Learning Task ^4^. Participants were presented a list of 15 words being read out five times (trial 1 -5), which participants had to recall each time. After an interference list of 15 new words, participants had to recall the first list again. After a 20-min break, in which other cognitive tasks were performed, participants had to recall the first list again. Finally, a list of 50 words containing all words from the first list, interference list and 20 completely new words was presented and participants had to indicate words from the first list. In accordance with Helmstaedter et al. (2001), we chose 3 out of 11 possible variables from the VLMT that resemble the core factors assessed by the VLMT. They included learning ability (best measured by the sum of the first 5 trials *(free recall trial)*, long-term consolidation (difference in performance between trial 5 and 7 *(loss after temporal delay)*) and long-term recognition performance (number of recognized words in the final list of 50 words after temporal delay *(cued recall trial)*).

*Attentional performance*

The Test of Attentional Performance (TAP) assesses cognitive domains related to attentional performance ^5^ containing 13 subtest out of which two were administered in this study to measure attentional performance: “alertness” and “divided attention”. The task alertness consists of two subsets, one with an auditory cue *(cued)* and one subset without any cue *(non-cued)*. Mean reaction time (in milliseconds; ms) from the cued and non-cued alertness trials were analyzed as measures of intrinsic alertness, which is considered a requirement for more complex cognitive functioning ^6^. Divided attention was assessed in a “dual-task” paradigm, in which auditory and visual stimuli had to be attended to simultaneously. We used mean reaction time (in ms) of auditory and visual targets for the current analysis.

*Inhibitory control*

During the “Go/NoGo” task of the TAP, participants were presented with a target stimulus (Go trial) that participants had to react to as fast as possible and a similar-looking distractor (NoGo trial) which they had to withhold response assessing inhibition ^7^. For purposes of better interpretation, we computed an inverse efficiency score (IES; Townsend & Ashby, 1978) with a higher IES representing lower inhibition competencies. Further, a number Stroop task and a Stop-Signal task ^9,10^ implemented using the Psychophysics Toolbox in MATLAB were administered. In the Stroop task, participants had to indicate how many digits were presented while ignoring their value. In the Stop-Signal Task, participants had to indicate whether an arrow was pointing to the left unless the arrow was followed by an upward-pointing arrow (the stop signal), in which case participants had to withhold their response. With the Stoop tasks requiring inhibition of an irrelevant attribute and the Stop-Signal tasks requiring inhibition of prepotent motor responses ^11^ we added these two tasks to the Go/NoGo to better assess inhibitory control.

The final analysis of cognitive testing included the following variables: 1) Stroop Task, 2) Stop-Signal Task, 3) TAP Go/NoGo, 4) Divided Attention - auditory, 5) Divided Attention - visual, 6) Alertness - cued, 7) Alertness - non-cued, 8) VLMT - free recall trial, 9) VLMT loss after temporal delay and 10) VLMT cued recall trial.

*Substance use*

Use of nicotine, alcohol, cannabis, MDMA, amphetamine, methamphetamine, cocaine, hallucinogens, ketamine, opiates and other substances was assessed in a personal interview (see Golub et al., 2021). Days and amount of use for the previous month and year were assessed in order to measure extent of substance use. Extent of substance use was measured as the average amount of use per usual consumption day multiplied with average frequency of use during previous year:

$$Extent of Substance use=Q \times F$$

Q = Average quantity per day of use during previous year in gram.

F = Frequency during previous year in days per month.

CCU was defined as at least weekly use of cannabis during the previous year and cannabis use related problems (e.g. loss of control of how much cannabis is used, craving, neglecting of responsibilities due to cannabis use, etc. see Golub et al. (2021) leading to presenting at the outpatient clinic). CCU-extent was defined as the extent of substance use of self-reported past year cannabis use.

*Qualitative measures of THC Consume*

Abstinence was monitored via urine screening (Multi 10TT Test Barcoded, Drug-Screen, nal von minden) before testing cognitive task performance, all individuals were required to be at least 24h abstinent when cognitive testing was performed. Participants that did not reach the cut-off for THC metabolite 11-nor-9-carboxy-Δ9-tetrahydrocannnabinol (THCCOOH) 50 ng/ml in urine were considered abstinent. In frequent users detection of THCCOOH is highly variable depending on amount of THCCOOH that was originally metabolized and BMI of the subject ^12^. However, the average of days of first negative test after abstinence was of users with initial values of >150 ng/mg was at least two weeks (15.4 days) in the study by Goodwin et al. (2008) so we considered participants with negative screening at least two weeks abstinent and participants with positive screening at least 24h abstinent due to self-reports.

*Statistical Analysis*

A Principal Component Analysis (PCA) was performed in R ^13^ using the princomp() function based on whole genome DNA methylation of leukocyte CpG sites after removing X/Y chromosomes to test whether there were differences in whole genome DNA methylation between adolescents with CCU and controls. A subsequent analysis of variances (ANOVA) was performed with SPSS (IBM SPSS Statistics for Windows, Version 25.0) with whole genome DNA methylation (mean beta values across all CpGs) as dependent variable and group (adolescents with CCU/controls) as independent variable. Effect sizes were defined after Cohen ^14^ as small (η^2^_part_ ≥ .01, *r* ≥ .1), medium (η^2^_part_ ≥ .06, *r* ≥ .3), and large effects (η^2^_part_ ≥ .14, *r* ≥ .5).

In an explorative approach, we first identified all relevant CpG sites and cognitive variables, both of which had to be associated with CCU-extent (Pearson correlations), before modelling and testing all mediations that were possible with the identified variables. Normal distribution was met for VLMT free recall trial. Beta values of the *k* = 6 identified CpGs, CCU-extent and VLMT cued recall trial were not normally distributed. Before identifying relevant CpG sites we ran a Multivariate Analysis of Variance (MANOVA) with cognitive tasks as dependent variable and group as independent variable to test whether there were differences between the groups. In order to identify relevant CpG sites, we performed correlation analyses using R and RStudio version 4.0.1 ^13^ between CCU-extent and CpGs site methylation (*k* = 866238). The significance level was set to 5.91^-8^ (Bonferroni correction) in order to control for alpha error inflation due to multiple testing. We repeated the correlation analyses in an extended sample after adding *n* = 5 patients with CCU and *n* = 6 controls that were matched on gender, age, depressive symptoms and number of co-occurring disorders in order to test the associations of CCU extent and CpG methylation on a larger sample. Further, in order to identify relevant cognitive variables, we performed *k* = 10 bivariate Pearson correlation analyses between variables of cognitive tasks and CCU-extent using SPSS. We defined a-priori that only those variables would be used in the next step that resulted in medium to large associations with CCU-extent and that significance level would not be considered the main criterion as it has previously being discussed to be lowered in explorative studies ^15^. As a-priori defined we ran mediation analyses for CCU-extent associated CpG sites and CCU-extent associated cognitive variables. Mediation analyses for *k* = 2 CCU-extent associated cognitive variables and *k* = 6 CCU-extent associated CpG sites were performed with PROCESS Macro (model 4, Preacher & Hayes, 2008). PROCESS provides both a significance test and an effect size estimate with 95% confidence interval for the mediational effect of mediator variable M (‘methylation of a specific CpG site’) on the relationship between a predictor X (‘CCU-extent’) and an outcome Y (‘cognitive task performance’). This ‘indirect effect’ (c’) describes by how much the relationship between X and Y (c) is affected by the relationship between X and M (a), and the relationship between M and Y (b). Alpha was set to .05 for mediation analyses.

We performed two posteriori analyses to exclude possible confounding variables. First, we were not able match the groups for tobacco and alcohol use frequency. Therefore, we performed the aforementioned *k* = 12 mediation analyses with frequency of tobacco and alcohol use as covariates to control for possible confounding influences of tobacco and alcohol use. Tobacco and alcohol use did not influence on the mediating effect of CpG methylation on the relationship between CCU-extent and verbal learning task performance, see Supplemental Information, Tables S1 – S4. Due to missing values in the tobacco and alcohol quantity-frequency variables, we originally used frequency variables for tobacco and alcohol as covariates in the posteriori mediation analyses. After that, we repeated the mediation analyses with quantity-frequency variables and found no differences between results of the mediation with frequency and the mediations with quantity-frequency, see Supplemental Information Tables S5-8.

Second, we performed an ANOVA with scores in VLMT free recall trial and VLMT cued recall trial as dependent variable and time since last THC exposure (dichotomous variable; adolescents that were abstinent from cannabis at least two weeks before the test /adolescents that consumed THC within last two weeks but not within the last 24h prior testing) as independent variable. We included this analysis in order to control for short term and possible withdrawal effects of cannabis use during weeks before the study and to focus on the effects of long-term cannabis use. There were no differences in VLMT trial 1-5 between current users (*M* = 51.75, *SD* = 10.75) and those who were currently abstinent (*M* = 49.8, *SD* = 14.8, *F* = 0.05, *p* = .833, η² = .01). Neither did scores in VLMT cued recall trial differ between current users (*M* = 13.8, *SD* = 1.9) and currently abstinent adolescents with CCU (*M* = 12.6, *SD* = 2.3, *F* = 0.64, *p* = .449, η² = .08).

Further, we performed binary regression analyses with gender as depended variable and cannabis exposition, scores in VLMT free recall and cued recall trial and the six identified genes as predictors. We added this analysis to exclude possible confounding influences of gender.

**Literature**

1. Golub, Y., Basedow, L. A., Meiron Zwipp, J., Kuitunen-Paul, S. & Roessner, V. *DELTA - Dresdner Multimodale Therapie für Jugendliche mit chronischem Suchtmittelkonsum*. (Hogrefe Verlag, 2021).

2. Fortin, J.-P., Triche, T. J., Jr & Hansen, K. D. Preprocessing, normalization and integration of the Illumina HumanMethylationEPIC array with minfi. *Bioinformatics* **33**, 558–560 (2017).

3. Helmstaedter, C., Lendt, M. & Lux, S. *VLMT: Verbaler Lern- und Merkfähigkeitstest*. (Beltz Test, 2001).

4. Bean, J. Rey Auditory Verbal Learning Test, Rey AVLT. in *Encyclopedia of Clinical Neuropsychology* (eds. Kreutzer, J. S., DeLuca, J. & Caplan, B.) 2174–2175 (Springer, 2011). doi:10.1007/978-0-387-79948-3_1153.

5. Fimm, B. & Zimmermann, P. *Testbatterie zur Aufmerksamkeitsprüfung: (TAP)*. (Psytest, 2002).

6. Sturm, W. Functional anatomy of intrinsic alertness: evidencefor a fronto-parietal-thalamic-brainstem network in theright hemisphere. *Neuropsychologia* **37**, 797–805 (1999).

7. Kiefer, M., Marzinzik, F., Weisbrod, M., Scherg, M. & Spitzer, M. The time course of brain activations during response inhibition: evidence from event-related potentials in a go/no go task. *NeuroReport* **9**, 765–770 (1998).

8. Townsend, J. & Ashby, F. Methods of modeling capacity in simple processing systems. in *Cognitive Theory* vol. 3 199–239 (1978).

9. Wolff, M., Krönke, K.-M., Venz, J., Kräplin, A., Bühringer, G., Smolka, M. N., *et al.* Action versus state orientation moderates the impact of executive functioning on real-life self-control. *J Exp Psychol Gen* **145**, 1635–1653 (2016).

10. Wolff, M., Enge, S., Kräplin, A., Krönke, K.-M., Bühringer, G., Smolka, M. N., *et al.* Chronic stress, executive functioning, and real-life self-control: An experience sampling study. *Journal of Personality* **89**, 402–421 (2021).

11. Portugal, A. C. A., Afonso Jr, A. S., Caldas, A. L., Maturana, W., Mocaiber, I. & Machado-Pinheiro, W. Inhibitory mechanisms involved in Stroop-matching and stop-signal tasks and the role of impulsivity. *Acta Psychologica* **191**, 234–243 (2018).

12. Goodwin, R. S., Darwin, W. D., Chiang, C. N., Shih, M., Li, S.-H. & Huestis, M. A. Urinary Elimination of 11-Nor-9-Carboxy- 9-tetrahydrocannnabinol in Cannabis Users During Continuously Monitored Abstinence. *Journal of Analytical Toxicology* **32**, 562–569 (2008).

13. R Core Team. R: A language and environment for statistical computing. R Foundation for Statistical Computing, Vienna, Austria. https://www.gbif.org/tool/81287/r-a-language-and-environment-for-statistical-computing (2018).

14. Cohen, J. *Statistical power analysis for the behavioral sciences*. (Psychology Press, 2009).

15. Lee, E. C. The statistical interpretation of pilot trials: should significance thresholds be reconsidered? *BMC Medical Research Methodology* **14:41**, 8 (2014).

Tables

**Table S1.** Mediating effects of CpG methylation for association between self-reported cannabis use and VLMT free recall trial with past years tobacco use frequency as covariate.

|  |  |  | *a* | | | *b* | | *c* | | *c‘* | Sobel Test | |
| --- | --- | --- | --- | --- | --- | --- | --- | --- | --- | --- | --- | --- |
| CpG site | *Gene* | Position  Assembly CRCh37/hg19 | *R²* | *b*^a^ [95% CI] | *R²* | | *b*^a^ [95% CI] | *R²* | b^a^ [95% CI] | *b*^a^ [95% CI] | Z | *p*_(Z)_ |
| Cg17285328 | *SH3PXD2B* | Chr5:171834167 | 90.7% | <-0.01  [<-0.01, <-0.01] | 47.9% | | 203.54  [50.87, 356.22] | 20.0% | -0.07  [-0.13, -0.01] | -0.21  [-0.41, 0.03] | -2.57 | -.010 |
| Cg20777378 | *-* | Chr3:184387942 | 88.7% | <-0.01  [<-0.01, <-0.01] | 39.0% | | 118.42  [31.70, 205.14] | 20.0% | -0.07  [-0.13, -0.01] | -0.16  [-0.35, 0.14] | -2.40 | .016 |
| Cg04904300 | *DGCR8* | Chr22:20076641 | 87.6% | <-0.01  [<-0.01, <-0.01] | 47.7% | | 122.92  [60.49, 185,39] | 20.0% | -0.07  [-0.13, -0.01] | -0.18  [-0.36, 0.01] | -3.45 | <.001 |
| Cg08923376 | *ZNF107* | Chr7:64149928 | 89.0% | <-0.01  [<-0.01, <-0.01] | 38.5% | | 78.40 [-12.94, 169.74] | 20.0% | -0.07  [-0.13, <-0.01] | -0.17  [-0.34, 0.06] | -1.76 | .079 |
| Cg04270414 | *SNX15; SAC3D1* | Chr11:64807235 | 86.9% | <-0.01  [<-0.01, <-0.01] | 44.1% | | 114.93  [32.46, 197.41] | 20.0% | -0.07  [-0.13, <-0.01] | -0.16  [-0.32, 0.08] | -2-48 | .013 |
| Cg23767840 | *EPN2* | Chr17:19174122 | 90.7% | <-0.01  [<-0.01, <-0.01] | 46.4% | | 100.29  [31.03, 169.56] | 20.0% | -0.07  [-0.13, -0.01] | -0.16  [-0.46, 0.03] | -2.53 | .011 |

*Note. ^a^ =* unstandardized coefficient b. *a* displays the relationship between predictor and mediator. *b* displays the relationship between mediator and outcome variable. *c* displays the relationship between predictor and outcome variable. *c’* displays the indirect predictor and mediator.

**Table S2.** Mediating effects of CpG methylation for association between self-reported cannabis use and VLMT cued recall trial with past years tobacco use frequency as covariate.

|  |  |  | *a* | | | *b* | | *c* | | *c‘* | Sobel Test | |
| --- | --- | --- | --- | --- | --- | --- | --- | --- | --- | --- | --- | --- |
| CpG site | *Gene* | Position  Assembly CRCh37/hg19 | *R²* | *b*^a^ [95% CI] | *R²* | | *b*^a^ [95% CI] | *R²* | *b*^a^ [95% CI] | *b*^a^ [95% CI] | Z | *p*_(Z)_ |
| Cg17285328 | *SH3PXD2B* | Chr5:171834167 | 90.8% | <-0.01  [<-0.01, <-0.01] | 43.1% | | 8.12  [-16.51, 32.75] | 41.5% | -0.02  [-0.02, -0.01] | <-0.01  [-0.04, 0.02] | -0.69 | .487 |
| Cg20777378 | - | Chr3:184387942 | 88.7% | <-0.01  [<-0.01, <-0.01] | 44.5% | | 7.71  [-8.94, 24,35] | 41.5% | -0.02  [-0.02, -0.01] | -0,01  [-0.04, 0.03] | -0.95 | .344 |
| Cg04904300 | *DGCR8* | Chr22:20076641 | 87,5% | <-0.01  [<-0.01, <-0.01] | 43.4% | | 5.36  [-12.64, 23,63] | 41.5% | -0.02  [-0.02, <-0.01] | <-0.01  [-0.03, 0.02] | -0.63 | .530 |
| Cg08923376 | *ZNF107* | Chr7:64149928 | 88,9% | <-0.01  [<-0.01, <-0.01] | 43.2% | | 3.99  [-15.9, 23.88] | 41.5% | -0.02  [-0.02, <-0.01] | <-0.01  [-0.04, 0.02] | -0.42 | .671 |
| Cg04270414 | *SNX15; SAC3D1* | Chr11:64807235 | 86,9% | <-0.01  [<-0.01, <-0.01] | 45.0% | | 7.29  [-7.62, 22.19] | 41.5% | -0.02  [-0.02, <-0.01] | -0.01  [-0.03, 0.02] | -1.00 | .316 |
| Cg23767840 | *EPN2* | Chr17:19174122 | 85,8% | <-0.01  [<-0.01, <-0.01] | 44.0% | | 5.09  [-8.61, 18,78] | 41.5% | -0.02  [-0.02, <-0.01] | <-0.01  [-0.04, 0.03] | -0.77 | .443 |

*Note. ^a^ =* unstandardized coefficient b. *a* displays the relationship between predictor and mediator. *b* displays the relationship between mediator and outcome variable. *c* displays the relationship between predictor and outcome variable. *c’* displays the indirect predictor and mediator.

**Table S3.** Mediating effects of CpG methylation for association between self-reported cannabis use and VLMT free recall trial with past years alcohol use frequency as covariate.

|  |  |  | *a* | | | *b* | | *c* | | *c‘* | Sobel Test | |
| --- | --- | --- | --- | --- | --- | --- | --- | --- | --- | --- | --- | --- |
| CpG site | *Gene* | Position  Assembly CRCh37/hg19 | *R²* | *b*^a^ [95% CI] | *R²* | | *b*^a^ [95% CI] | *R²* | *b*^a^ [95% CI] | *b*^a^ [95% CI] | Z | *p*_(Z)_ |
| Cg17285328 | *SH3PXD2B* | Chr5:171834167 | 93.0% | <-0.01  [<-0.01, <0.01] | 45.6% | | 241.39 [-23.79, 506.58] | 15.8% | 241.39 [-23.79, 506.58] | 241.39 [-23.79, 506.58] | -1.00 | .317 |
| Cg20777378 | - | Chr3:184387942 | 90.8% | <-0.01  [<-0.01, <0.01] | 36.6% | | 137.10 [-81.47, 355.67] | 15.8% | 137.10 [-81.47, 355.67] | 137.10 [-81.47, 355.67] | -1.00 | .313 |
| Cg04904300 | *DGCR8* | Chr22:20076641 | 89.7% | <-0.01  [<-0.01, <0.01] | 43.1% | | 134.03  [-6.96, 275.02] | 15.8% | 134.03  [-6.96, 275.02] | 134.03  [-6.96, 275.02] | -1.00 | .317 |
| Cg08923376 | *ZNF107* | Chr7:64149928 | 87.4% | <-0.01  [<-0.01, <0.01] | 40.0% | | 84.15 [-33.61, 201.91] | 15.8% | 84.15 [-33.61, 201.91] | 84.15 [-33.61, 201.91] | -0.70 | .485 |
| Cg04270414 | *SNX15; SAC3D1* | Chr11:64807235 | 90.4% | <-0.01  [<-0.01, <0.01] | 39.1% | | 131.93 [-42.89, 306.75] | 15.8% | 131.93 [-42.89, 306.75] | 131.93 [-42.89, 306.75] | -0.88 | .379 |
| Cg23767840 | *EPN2* | Chr17:19174122 | 88.8% | <-0.01  [<-0.01, <0.01] | 42.6% | | 113.87 [-41.06, 268.79] | 15.8% | 113.87 [-41.06, 268.79] | 113.87 [-41.06, 268.79] | -0.91 | .364 |

*Note. ^a^ =* unstandardized coefficient b. *a* displays the relationship between predictor and mediator. *b* displays the relationship between mediator and outcome variable. *c* displays the relationship between predictor and outcome variable. *c’* displays the indirect predictor and mediator.

**Table S4.** Mediating effects of CpG methylation for association between self-reported cannabis use and VLMT cued recall trial with past years alcohol use frequency as covariate.

|  |  |  | *a* | | | *b* | | *c* | | *c‘* | Sobel Test | |
| --- | --- | --- | --- | --- | --- | --- | --- | --- | --- | --- | --- | --- |
| CpG site | *Gene* | Position  Assembly CRCh37/hg19 | *R²* | *b*^a^ [95% CI] | *R²* | | *b*^a^ [95% CI] | *R²* | *b*^a^ [95% CI] | *b*^a^ [95% CI] | Z | *p*_(Z)_ |
| Cg17285328 | *SH3PXD2B* | Chr5:171834167 | 93.0% | <-0.01  [<-0.01, <0.01] | 38.9% | | <-0.01  [<-0.01, <0.01] | 36.5% | <-0.01  [<-0.01, <0.01] | <-0.01  [<-0.01, <0.01] | -0.41 | .684 |
| Cg20777378 | - | Chr3:184387942 | 90.8% | <-0.01  [<-0.01, <0.01] | 41.4% | | <-0.01  [<-0.01, <0.01] | 36.5% | <-0.01  [<-0.01, <0.01] | <-0.01  [<-0.01, <0.01] | -0.47 | .636 |
| Cg04904300 | *DGCR8* | Chr22:20076641 | 89.7% | <-0.01  [<-0.01, <0.01] | 38.9% | | <-0.01  [<-0.01, <0.01] | 36.5% | <-0.01  [<-0.01, <0.01] | <-0.01  [<-0.01, <0.01] | -0.24 | .810 |
| Cg08923376 | *ZNF107* | Chr7:64149928 | 78.4% | <-0.01  [<-0.01, <0.01] | 40.6% | | <-0.01  [<-0.01, <0.01] | 36.5% | <-0.01  [<-0.01, <0.01] | <-0.01  [<-0.01, <0.01] | -0.37 | .708 |
| Cg04270414 | *SNX15; SAC3D1* | Chr11:64807235 | 90.4% | <-0.01  [<-0.01, <0.01] | 40.7% | | <-0.01  [<-0.01, <0.01] | 36.5% | <-0.01  [<-0.01, <0.01] | <-0.01  [<-0.01, <0.01] | -0.37 | .712 |
| Cg23767840 | *EPN2* | Chr17:19174122 | 88.9% | <-0.01  [<-0.01, <0.01] | 39.7% | | <-0.01  [<-0.01, <0.01] | 36.5% | <-0.01  [<-0.01, <0.01] | <-0.01  [<-0.01, <0.01] | -0.26 | .798 |

*Note. ^a^ =* unstandardized coefficient b. *a* displays the relationship between predictor and mediator. *b* displays the relationship between mediator and outcome variable. *c* displays the relationship between predictor and outcome variable. *c’* displays the indirect predictor and mediator.

**Table S5.** Mediating effects of CpG methylation for association between self-reported cannabis use and VLMT free recall trial with past years tobacco use quantity frequency index as covariate.

|  |  |  | *a* | | | *b* | | *c* | | *c‘* | Sobel Test | |
| --- | --- | --- | --- | --- | --- | --- | --- | --- | --- | --- | --- | --- |
| CpG site | *Gene* | Position  Assembly CRCh37/hg19 | *R²* | *b*^a^ [95% CI] | *R²* | | *b*^a^ [95% CI] | *R²* | *b*^a^ [95% CI] | *b*^a^ [95% CI] | Z | *p*_(Z)_ |
| Cg17285328 | *SH3PXD2B* | Chr5:171834167 | 93.0% | <-0.01  [<-0.01, <-0.01] | 62.0% | | 306.95  [148.61, 465.30] | 11.8% | -0.04  [-0.25, 0.16] | -0.23  [-0.48, 0.04] | -2.14 | .032 |
| Cg20777378 | - | Chr3:184387942 | 88.6% | <-0.01  [<-0.01, <0.01] | 38.6% | | 137.61  [-2.23, 277.44] | 11.8% | -0.04  [-0.25, -0.16] | -0.15  [-0.51, 0.15] | -1.30 | .192 |
| Cg04904300 | *DGCR8* | Chr22:20076641 | 88.1% | <-0.01  [<-0.01, <0.01] | 44.0% | | 134.15  [76.54, 191,76] | 11.8% | -0.04  [-0.25, 0.16] | -0.02  [-0.47, 0.03] | -1.67 | .094 |
| Cg08923376 | *ZNF107* | Chr7:64149928 | 87.4% | <-0.01  [<-0.01, <0.01] | 38.4% | | 86.86 [-35.76, 209.48] | 11.8% | -0.04  [-0.25, 0.16] | -0.18  [-0.50, 0.06] | -1.08 | .281 |
| Cg04270414 | *SNX15; SAC3D1* | Chr11:64807235 | 88.6% | <-0.01  [<-0.01, <0.01] | 44.1% | | 141.19  [62.86, 219.51] | 11.8% | -0.04  [-2.48, 0.16] | -0.14  [-0.47, 0.11] | -1.48 | .138 |
| Cg23767840 | *EPN2* | Chr17:19174122 | 86.6% | <-0.01  [<-0.01, <0.01] | 44.6% | | 113.54  [53.94, 173.14] | 11.8% | -0.04  [-0.25, 0.16] | -0.14  [-0.57, 0.04] | -1.39 | .164 |

*Note. ^a^ =* unstandardized coefficient b. *a* displays the relationship between predictor and mediator. *b* displays the relationship between mediator and outcome variable. *c* displays the relationship between predictor and outcome variable. *c’* displays the indirect predictor and mediator.

**Table S6.** Mediating effects of CpG methylation for association between self-reported cannabis use and VLMT cued recall trial temporal delay with past years tobacco use quantity frequency index as covariate.

|  |  |  | *a* | | | *b* | | *c* | | *c‘* | *Sobel Test* | |
| --- | --- | --- | --- | --- | --- | --- | --- | --- | --- | --- | --- | --- |
| CpG site | *Gene* | Position  Assembly CRCh37/hg19 | *R²* | *b*^a^ [95% CI] | *R²* | | *b*^a^ [95% CI] | *R²* | *b*^a^ [95% CI] | *b*^a^ [95% CI] | Z | *p*_(Z)_ |
| Cg17285328 | *SH3PXD2B* | Chr5:171834167 | 93.0% | <-0.01  [<-0.01, <-0.01] | 49.4% | | 17.72  [-13.94, 49.37] | 43.5% | <0.01  [-0.01, 0.02] | -0.01  [-0.03, 0.02] | -1.03 | .302 |
| Cg20777378 | - | Chr3:184387942 | 88.6% | <-0.01  [<-0.01, <0,01] | 49.9% | | 11.34  [-10.07, 32,76] | 43.5% | -0.02  [-0.05, <0.01] | -0,01  [-0.04, 0.03] | -0.88 | .379 |
| Cg04904300 | *DGCR8* | Chr22:20076641 | 88,1% | <-0.01  [<-0.01, <0.01] | 48.9% | | 9.20  [-9.28, 27,68] | 43.5% | -0.02  [-0.05, <0.01] | -0.01  [-0.04, 0.02] | -0.84 | .404 |
| Cg08923376 | *ZNF107* | Chr7:64149928 | 87,4% | <-0.01  [<-0.01, <0.01] | 46.8% | | 5.17  [-12.84, 23.17] | 43.5% | -0.02  [-0.05, <0.01] | -0.01  [-0.04, 0.02] | -0.52 | .603 |
| Cg04270414 | *SNX15; SAC3D1* | Chr11:64807235 | 88,6% | <-0.01  [<-0.01, <0.01] | 51.2% | | 11.51  [-3.26, 26.29] | 43.5% | -0.02  [-0.05, <0.01] | -0.01  [-0.04, 0.02] | -1.09 | .277 |
| Cg23767840 | *EPN2* | Chr17:19174122 | 86,6% | <-0.01  [<-0.01, <0.01] | 49.81% | | 8.38  [-5.21, 21,97] | 43.5% | -0.02  [-0.05, <0.01] | -0.01  [-0.05, 0.02] | -0.90 | .369 |

*Note. ^a^ =* unstandardized coefficient b. *a* displays the relationship between predictor and mediator. *b* displays the relationship between mediator and outcome variable. *c* displays the relationship between predictor and outcome variable. *c’* displays the indirect predictor and mediator.

**Table S7.** Mediating effects of CpG methylation for association between self-reported cannabis use and VLMT free recall trial with past years alcohol use quantity frequency index as covariate.

|  |  |  | *a* | | | *b* | | *c* | | *c‘* | Sobel Test | |
| --- | --- | --- | --- | --- | --- | --- | --- | --- | --- | --- | --- | --- |
| CpG site | *Gene* | Position  Assembly CRCh37/hg19 | *R²* | *b*^a^ [95% CI] | *R²* | | *b*^a^ [95% CI] | *R²* | *b*^a^ [95% CI] | *b*^a^ [95% CI] | Z | *p*_(Z)_ |
| Cg17285328 | *SH3PXD2B* | Chr5:171834167 | 93.0% | <-0.01  [<-0.01, <0.01] | 45.6% | | 241.39 [-23.79, 506.58] | 15.8% | 241.39 [-23.79, 506.58] | 241.39 [-23.79, 506.58] | -1.00 | .317 |
| Cg20777378 | - | Chr3:184387942 | 90.8% | <-0.01  [<-0.01, <0.01] | 36.6% | | 137.10 [-81.47, 355.67] | 15.8% | 137.10 [-81.47, 355.67] | 137.10 [-81.47, 355.67] | -1.00 | .313 |
| Cg04904300 | *DGCR8* | Chr22:20076641 | 89.7% | <-0.01  [<-0.01, <0.01] | 43.1% | | 134.03  [-6.96, 275.02] | 15.8% | 134.03  [-6.96, 275.02] | 134.03  [-6.96, 275.02] | -1.00 | .317 |
| Cg08923376 | *ZNF107* | Chr7:64149928 | 87.4% | <-0.01  [<-0.01, <0.01] | 40.0% | | 84.15 [-33.61, 201.91] | 15.8% | 84.15 [-33.61, 201.91] | 84.15 [-33.61, 201.91] | -0.70 | .485 |
| Cg04270414 | *SNX15; SAC3D1* | Chr11:64807235 | 90.4% | <-0.01  [<-0.01, <0.01] | 39.1% | | 131.93 [-42.89, 306.75] | 15.8% | 131.93 [-42.89, 306.75] | 131.93 [-42.89, 306.75] | -0.88 | .379 |
| Cg23767840 | *EPN2* | Chr17:19174122 | 88.8% | <-0.01  [<-0.01, <0.01] | 42.6% | | 113.87 [-41.06, 268.79] | 15.8% | 113.87 [-41.06, 268.79] | 113.87 [-41.06, 268.79] | -0.91 | .364 |

*Note. ^a^ =* unstandardized coefficient b. *a* displays the relationship between predictor and mediator. *b* displays the relationship between mediator and outcome variable. *c* displays the relationship between predictor and outcome variable. *c’* displays the indirect predictor and mediator.

**Table S8.** Mediating effects of CpG methylation for association between self-reported cannabis use and VLMT cued recall trial with past years alcohol use quantity frequency index as covariate.

|  |  |  | *a* | | | *b* | | *c* | | *c‘* | Sobel Test | |
| --- | --- | --- | --- | --- | --- | --- | --- | --- | --- | --- | --- | --- |
| CpG site | *Gene* | Position  Assembly CRCh37/hg19 | *R²* | *b*^a^ [95% CI] | *R²* | | *b*^a^ [95% CI] | *R²* | *b*^a^ [95% CI] | *b*^a^ [95% CI] | Z | *p*_(Z)_ |
| Cg17285328 | *SH3PXD2B* | Chr5:171834167 | 93.0% | <-0.01  [<-0.01, <0.01] | 38.9% | | <-0.01  [<-0.01, <0.01] | 36.5% | <-0.01  [<-0.01, <0.01] | <-0.01  [<-0.01, <0.01] | -0.41 | 0.68 |
| Cg20777378 | - | Chr3:184387942 | 90.8% | <-0.01  [<-0.01, <0.01] | 41.4% | | <-0.01  [<-0.01, <0.01] | 36.5% | <-0.01  [<-0.01, <0.01] | <-0.01  [<-0.01, <0.01] | -0.47 | 0.64 |
| Cg04904300 | *DGCR8* | Chr22:20076641 | 89.7% | <-0.01  [<-0.01, <0.01] | 38.9% | | <-0.01  [<-0.01, <0.01] | 36.5% | <-0.01  [<-0.01, <0.01] | <-0.01  [<-0.01, <0.01] | -0.24 | 0.81 |
| Cg08923376 | *ZNF107* | Chr7:64149928 | 78.4% | <-0.01  [<-0.01, <0.01] | 40.6% | | <-0.01  [<-0.01, <0.01] | 36.5% | <-0.01  [<-0.01, <0.01] | <-0.01  [<-0.01, <0.01] | -0.37 | 0.71 |
| Cg04270414 | *SNX15; SAC3D1* | Chr11:64807235 | 90.4% | <-0.01  [<-0.01, <0.01] | 40.7% | | <-0.01  [<-0.01, <0.01] | 36.5% | <-0.01  [<-0.01, <0.01] | <-0.01  [<-0.01, <0.01] | -0.37 | 0.71 |
| Cg23767840 | *EPN2* | Chr17:19174122 | 88.9% | <-0.01  [<-0.01, <0.01] | 39.7% | | <-0.01  [<-0.01, <0.01] | 36.5% | <-0.01  [<-0.01, <0.01] | <-0.01  [<-0.01, <0.01] | -0.26 | 0.80 |

*Note. ^a^ =* unstandardized coefficient b. *a* displays the relationship between predictor and mediator. *b* displays the relationship between mediator and outcome variable. *c* displays the relationship between predictor and outcome variable. *c’* displays the indirect predictor and mediator

|  | **Total**  ***n* = 9** | **Abstinent**  ***n* = 5** | **Not abstinent**  ***n* = 4** | **abstinent group vs. not abstinent group** | | |
| --- | --- | --- | --- | --- | --- | --- |
|  |  | *M* (SD)/*N* (%) | | *F*(1,15) | *p* | η^2^_part_ |
| VLMT free recall trial | 50,67 (12,43) | 49.80 (14.84) | 51.75 (10.75) | 0.05 | .833 | .01 |
| VLMT cued recall trial | 13,11 (2,09) | 12.60 (2.30) | 13.75 (1.89) | 0.64 | 449 | .08 |

**Table S9.** Results of group differences between adolescents with CCU being abstinent from cannabis during the previous weeks and those not being abstinent but at least being 24h abstinent, for *n* = 9.

**Table S10.** Pearson correlations between *k* = 6 identified CpGs and CCU-extent in the extended sample.

| CpG site | *Gene* | Position  Assembly CRCh37/hg19 | *correlation coefficients r and p-values (p) of CCU extent and beta values* |
| --- | --- | --- | --- |
| Cg17285328 | SH3PXD2B | Chr5:171834167 | -.71 (1.51E-05) |
| Cg20777378 | - | Chr3:184387942 | -.68 (5.79E-05) |
| Cg04904300 | DGCR8 | Chr22:20076641 | -.66 (9.37E-05) |
| Cg08923376 | ZNF107 | Chr7:64149928 | -.55 (.002) |
| Cg04270414 | SNX15; SAC3D1 | Chr11:64807235 | -.52 (.004) |
| Cg23767840 | EPN2 | Chr17:19174122 | -.52 (.004) |

**Table S11.** Binary Regression analysis of CCU-extent, scores in VLMT free recall and cued recall trial and CpG methylation in Cg17285328 (*SH3PXD2B*), Cg20777378 (-), Cg04904300 (*DGCR8*), Cg08923376 (*ZNF107*), Cg04270414 (*SNX15;SAC3D1*) and Cg23767840 (*EPN2*) for n = 18 individuals.

|  | **Total**  *n* = 18 | **Male**  *n* = 14 | **Female**  *n* = 4 | ***B*** | ***p*** | ***OR*** | **[95%C*I*]** |
| --- | --- | --- | --- | --- | --- | --- | --- |
| CCU extent | 39.5 (79.4) | 32.2 (79.4) | 180 (64.7) | 0.05 | .476 | 1.01 | [0.99; 1.02] |
| VLMT free recall trial | 49.5 (10) | 49.1 (9.4) | 64 (51) | 0.11 | .234 | 1.12 | [0.93; 1.35] |
| VLMT cued recall trial | 13.4 (1.6) | 13.6 (1.7) | 15 (12.8) | -0.82 | .174 | 0.44 | [0.13; 1.44] |
| Cg17285328 *(SH3PXD2B)* | 0.9 (0.1) | 0.9 (0.1) | 0.9 (0.9) | 15673.67 | .984 | <0.01 | [<0.01; <0.01] |
| Cg20777378 *(-)* | 0.8 (0.1) | 0.8 (0.1) | 0.9 (0.8) | -565.16 | .995 | <0.01 | [<0.01; <0.01] |
| Cg04904300 *(DGCR8)* | 0.9 (0.1) | 0.9 (0.1) | 0.9 (0.8) | -17402.45 | .985 | <0.01 | [<0.01; <0.01] |
| Cg08923376 *(ZNF107)* | 0.9 (0.2) | 0.9 (0.2) | 0.9 (0.8) | 1677.66 | .985 | <0.01 | [<0.01; <0.01] |
| Cg04270414 *(SNX15;SAC3D1)* | 0.9 (0.1) | 0.9 (0.1) | 0.9 (0.8) | -58495.33 | .984 | <0.01 | [<0.01; <0.01] |
| Cg23767840 *(EPN2)* | 0.9 (0.1) | 0.9 (0.1) | 0.9 (0.8) | 53837.90 | .984 | <0.01 | [<0.01; <0.01] |
